# Metastasis of distal esophageal carcinoma to the thyroid with presentation simulating primary thyroid carcinoma: a case report and review of the literature

**DOI:** 10.1186/1477-7819-12-106

**Published:** 2014-04-23

**Authors:** En-dong Chen, Pu Cheng, Xing-qiang Yan, Yun-liang Ye, Cheng-ze Chen, Xiu-huan Ji, Xiao-hua Zhang

**Affiliations:** 1 Department of Surgical Oncology, The First Affiliated Hospital of Wenzhou Medical University, Wenzhou, Zhejiang, People’s Republic of China; 2 Department of Pathology, The First Affiliated Hospital of Wenzhou Medical University, Wenzhou, Zhejiang, People’s Republic of China

**Keywords:** Thyroid metastasis, esophageal neoplasms, squamous cell carcinoma

## Abstract

Metastasis to the thyroid is extremely rare. There is a lack of awareness of and adequate preparation for this situation, especially in an individual without a past history of malignancy. We describe a rare case of a 61-year-old man in whom a primary distal esophageal carcinoma gave rise to a metastatic palpable mass in the thyroid gland. Palliative bilateral near-total thyroidectomy was performed with pathology showing squamous cell carcinoma and tracheostomy was carried out simultaneously due to airway compression with related symptoms. A review of the literature only reveals 4 similar cases. Secondary neoplasm of the thyroid mimicking a primary malignant lesion is seldom encountered, however, in order to make appropriate treatment, the most critical problem is to distinguish the difference between the above two and the final diagnosis can only be confirmed on pathologic examination. Although the prognosis of thyroid metastasis is commonly felt to be poor, improvement of living quality and prolongation of survival may be obtained in such patients through correct diagnosis and treatment.

## Background

Despite being a highly vascularized organ, the thyroid gland is an unusual site of clinically detectable cancer metastasis. According to reports, the incidence of intrathyroid metastases (ITM) in autopsy series varies from 1.25% to 24.0% in cancer patients [1,2].The most frequently noted primary sites are the kidney, breast, and lung [3-6]. The metastatic spread of gastrointestinal malignancies to the thyroid gland is relatively rare, and the majority come from the colo-rectum [7]. ITM originating from the alimentary tract are quite unusual based on our previous experience. In clinical practice, metastatic involvement of the thyroid is usually characterized by an indolent growth pattern, which results in few visible indications of the illness and difficulty in making a differential diagnosis from other primary thyroid neoplasms [8]. Thyroid metastasis from the esophagus has only been reported in four cases in the English literature [9-12]. Herein, a further case is reported of metastasis of esophageal carcinoma to the thyroid, which was misdiagnosed in the first place as primary thyroid carcinoma with unilateral lymph node metastasis, and a systematic literature review shows that this is the first case of this rare condition to be described in eastern China.

## Case presentation

In July 2011, a 61-year-old man presented to our department complaining of dyspnea, mild dysphagia and hoarseness for a few months with exacerbation for 10 days. There was no remarkable past medical, surgical, or family history. On admission, a large nodular mass on the left lobe of the thyroid was readily recognizable and was irregular, hard, immobile and painless on palpation. Thyroid sonogram findings demonstrated a 6.1 cm × 3.9 cm mass in the left lobe with low and heterogeneous echo and an enlarged cervical lymph node measuring 2.5 cm × 1.8 cm on the left side of the neck in the level-III region with hypoechogenicity. Fine-needle aspiration cytology of the thyroid disclosed diffuse infiltration of atypical cells with a high suspicion of malignancy, therefore the patient was scheduled for surgery.

**Figure 1:**
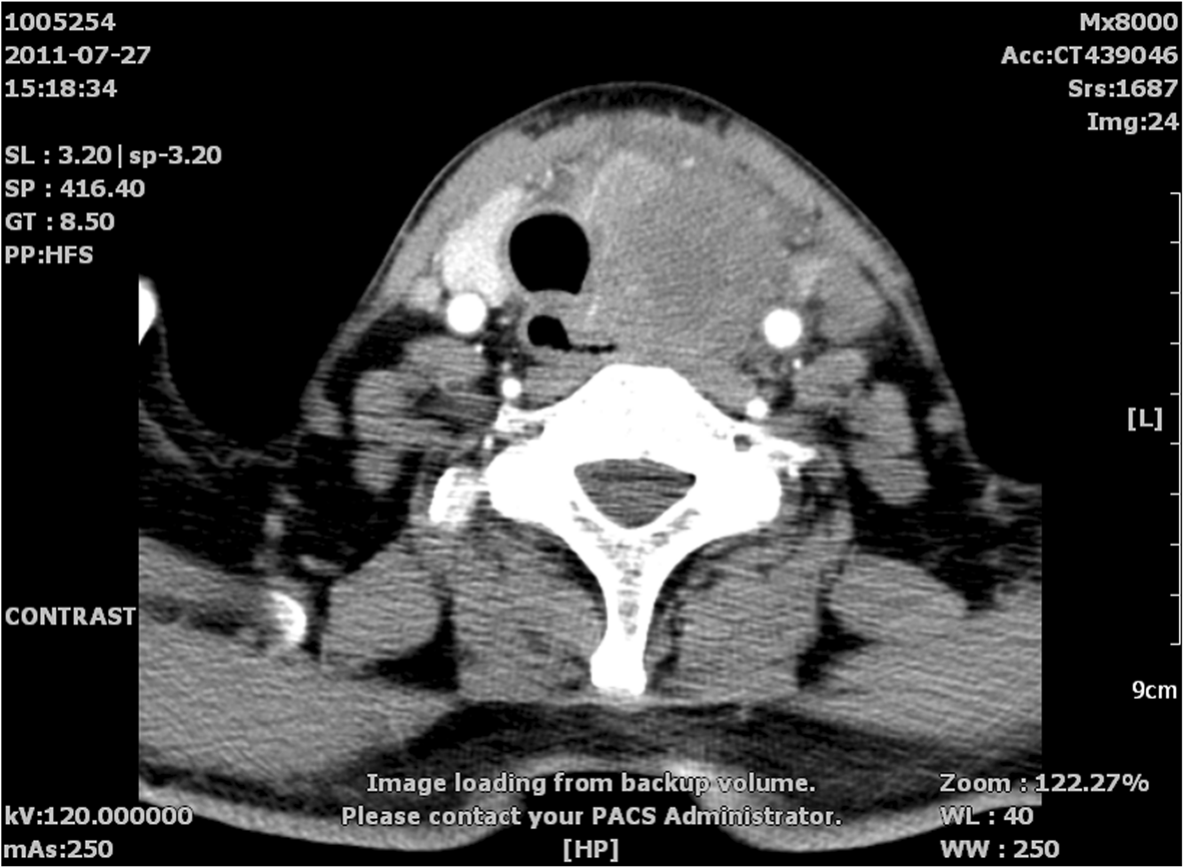
Neck contrast-enhanced computed tomography reveals a mass encasing the left carotid sheath vessels, esophagus, and trachea in the left thyroid lobe.

**Figure 2:**
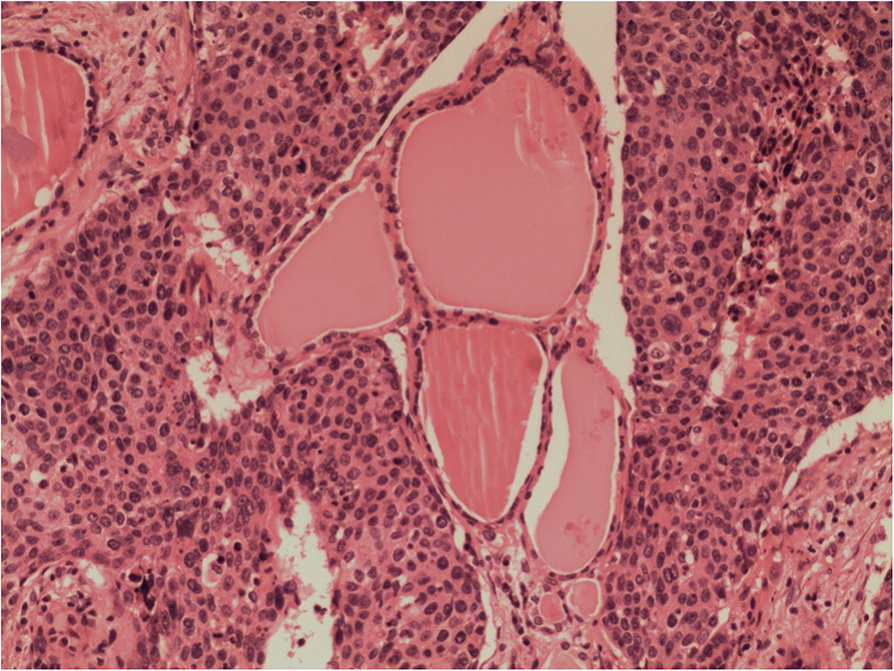
**Multi-focal nests of tumors cells are distributed nearby the follicles.** Hematoxylin and eosin staining, ×100.

**Figure 3:**
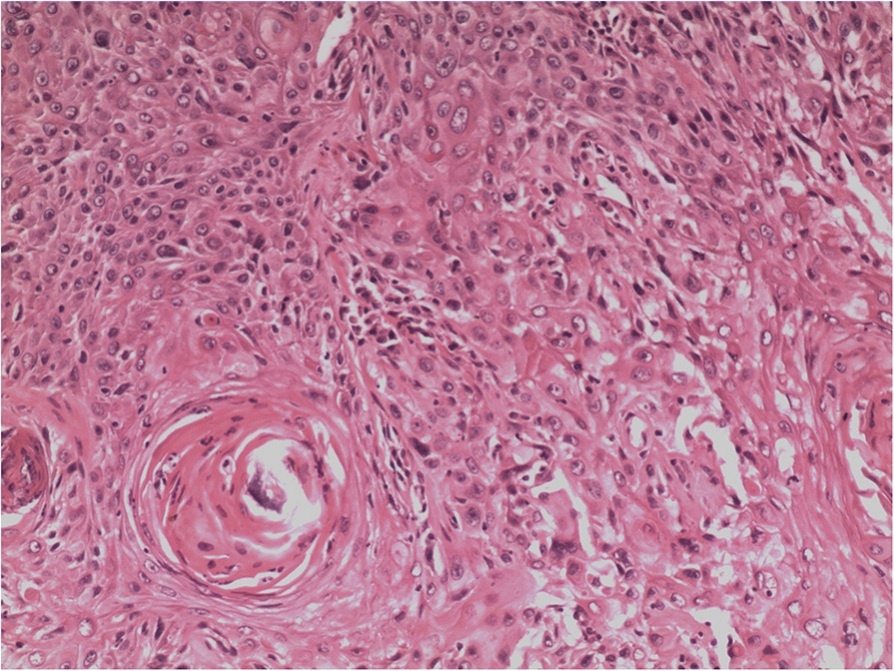
**Tumor cells originate from the esophagus.** Hematoxylin and eosin staining, ×200.

Chest radiography and laboratory examinations including thyroid hormone showed no abnormal findings. Blood calcium level was also checked for the possibility of medullary thyroid cancer, and was within normal limits. Neck contrast-enhanced computed tomography revealed a mass encasing the left carotid sheath vessels, esophagus, and trachea in the left thyroid lobe (Figure 1). The man underwent bilateral near-total thyroidectomy and tracheostomy for palliative purposes because adhesions to adjacent structures and the aggravation of dyspnea were observed pre- and intra-operatively. Histological examination from the resected specimen verified moderate differentiated squamous cell carcinoma (SCC) of the thyroid gland (Figure 2) with metastasis to the level-III region of the left cervical lymph nodes and characteristics similar to the esophageal lesions. Further workup with introscopy biopsy revealed a squamous cell carcinoma of the distal esophagus (Figure 3). One month post surgery, the patient started chemotherapy with a docetaxel and cisplatin (DC) regimen, and radiotherapy according to the 2011 National Comprehensive Cancer Network (NCCN) Esophageal Cancer Guidelines. Although the patient’s condition was kept under control initially, after five cycles of chemotherapy, his illness grew worse as the cancer disseminated to the peritoneal and mediastinal lymph nodes. Finally, eleven months after the diagnosis of metastasis, the patient died due to advanced esophageal carcinoma complicated by pneumonia and sepsis.

## Discussion

The incidence of metastatic spread of gastrointestinal malignancies to the thyroid gland is relatively low and most of them are from the colo-rectum [7]. ITM originating from the gastro-esophagus is poorly documented. A review of the English-language literature, searching for patients with secondary cancerous growth in the thyroid formed by transmission of tumor cells from primary esophageal and gastric carcinoma disclosed a total of four [9-12] and five reported cases [7,13-16], respectively. This article presents an additional case of thyroid metastasis from esophageal carcinoma occurring in a Chinese man and reviews the related references.

Table 1 summarizes the clinical circumstances for the nine cases previously published plus our report of thyroid metastasis from esophageal cancer. The age of the five female and five male patients at presentation was variable, ranging from 32 to 74 years with an average age of 61.5 years. The majority of patients underwent thyroidectomy (specific types of surgery are presented in Table 1: this was unknown in four cases). In the postoperative histopathological specimen, there were four patients whose thyroid cancer seeded from squamous cell carcinoma, two originated from the undifferentiated carcinoma with signet-ring cells, two were derived from poorly differentiated carcinoma with characteristics similar to the gastric lesions, and the remaining cases were from poorly differentiated adenocarcinomas. Of the patients with thyroid metastasis, most had a poor outcome and died shortly after the original diagnosis (specific survival times are presented in Table 1).

**Table 1:** Clinical data of patients with metastatic involvement of the thyroid

**Source**	**Age (yr)**	**Sex**	**Site of primary lesion**	**Type of secondary thyroid surgery**	**Pathology results from thyroid specimen**	Outcomes (months)*
Present case	61	M	Esophagus	Palliative bilateral NT + tracheostomy	SCC	11
Shuangshoti S *et al*. 1982 [9]	58	M	Esophagus	TT + ipsilateral CL	SCC	5
Yamada T *et al*. 1999 [10]	74	F	Esophagus	ST + Bilateral CL	SCC	/
Basu S *et al*. 2005 [11]	55	F	Esophagus	NA	SCC	NA
Cumbo-Nacheli G *et al*. 2007 [12]	32	M	Esophagus	NA	Poorly differentiated adenocarcinoma	NA
Yoshida A *et al*. 1989 [7]	71	M	Stomach	ST	Poorly differentiated adenocarcinoma	7
Ok E *et al*. 2000 [13]	60	F	Stomach	Bilateral ST	Undifferentiated carcinoma (with signet-ring cells)	1.5
Lee HC *et al*. 2010 [15]	71	M	Stomach	Bilateral NT	Poorly differentiated carcinoma	4
Ihn MH *et al*. 2009 [14]	63	F	Stomach	NA	Undifferentiated carcinoma (with signet-ring cells)	6
Poiana C *et al*. 2011 [16]	70	F	Stomach	NA	Poorly differentiated neuroendocrine carcinoma (with small cells)	NA

*Follow up since diagnosis of intrathyroid metastases; NA, no data available; NT, near-total thyroidectomy; ST, subtotal thyroidectomy; TT, total thyroidectomy; CL, cervical lymphadenectomy.

Table 2 summarizes the diagnosis of thyroid cancer for reported cases. Three patients had multifocal or widespread masses and all the others had only a solitary mass. Five patients were confirmed with unilateral or bilateral cervical lymph nodes metastases and two patients had no evidence of cervical lymphadenopathy (there was no reference to this for the remaining cases. Several patients received additional examinations (specific investigations are presented in Table 2).

**Table 2:** The diagnosis of thyroid cancer for reported cases

**Source**	**Tumor size (cm)**	**Ultrasound description**	**Extent of metastatic thyroid gland involvement**	**Cervical lymph nodes metastasis**
Present case	6.1 × 3.9	Heterogeneous, hypo-echoic	Left lobe, solitary mass	Left level-III region
Shuangshoti S *et al*. 1982 [9]	1.5 × 1.5	NA	Right lobe, solitary mass	Right level-II, −III and -IV region
Yamada T *et al*. 1999 [10]	NA	Calcified	Widespread masses; not specified	Bilateral level-II, −III and -IV region
Basu S *et al*. 2005 [11]	6 × 4	Irregular, hypo-echoic	Right lobe, solitary mass	Right level-III and -IV region
Cumbo-Nacheli G *et al*. 2007 [12]	2.5 × 2.8	NA	Right lobe, solitary mass	Right level-II, −III and -IV region
Yoshida A *et al*. 1989 [7]	10 × 4	NA	Left lobe and the isthmus, solitary mass	No evidence of disease
Ok E *et al*. 2000 [13]	4 × 5	NA	Right lobe, solitary mass	No evidence of disease
Lee HC *et al*. 2010 [15]	NA	NA	Bilateral, multifocal masses	NA
Ihn MH *et al*. 2009 [14]	NA	Diffuse, enlarged	Widespread masses; not specified	NA
Poiana C *et al*. 2011 [16]	NA	NA	Left lobe and the isthmus, solitary mass	No evidence of disease

NA, no data available.

Generally, despite being second only to the adrenal glands as the most vascular perfused organ in the body [17], the thyroid is rarely considered to be the sole site of metastases in the clinical setting and is usually asymptomatic [8,18]. Cichon *et al.* reported that metastasis to the thyroid only accounts for 2% to 3% of all thyroid carcinomas identified in the clinical setting [19]. The most common primary sites are the kidney, breast, and lung (see related references [3-6]). To the best of our knowledge there is little information to date in the English literature about thyroid metastasis from the esophagus, except for four published cases [9-12].

One question is raised by our case: how to prove that the SCC of the thyroid in our patient originated from a primary esophageal carcinoma? In general, it is difficult and challenging to accurately distinguish between primary and secondary neoplasm in the thyroid. Primary SCC rarely arise from the thyroid gland, especially in older patients with a long-standing history of goître [20,21]. The etiology remains mysterious and unclear, and presumed to originate from the metaplastic glandular epithelium. Fine-needle aspiration cytology is often used to obtain tissues for diagnosis. Nevertheless, its value in the discrimination between primary and metastatic thyroid malignancies is still uncertain when highly anaplastic cells are observed microscopically [2]. Our patient had no particular history of thyroid disease and the thyroid mass grew rapidly in a few months. What is more, the patient suffered an exacerbation of dyspnea. An urgent thyroidectomy was necessary to relieve threatening airway obstruction caused by tracheal compression. Postoperative histopathological examination revealed multi-focal lesions with similar pathological profiles to the esophageal lesions. Thorough work up reavealed a squamous carcinoma of the distal esophagus on esophagogastroduodenoscopy biopsy. Other findings included: dysphagia, dyspnea, hoarseness, vascular infiltration of SCC under the microscope, and some imaging observations. It was therefore confirmed as a primary esphageal cancer with metastasis to the thyroid.

Direct extension of adjacent primaries, a hematogenous pathway and lymphatic route for metastatic spread to the thyroid have been suggested [11,22]. Czech *et al.* suggested that the vertebral vein plexus may play an important role in the process of metastases from other organs to the thyroid [5]. Unfortunately, according to a review of the related literature, there is no reported case of careful imaging and pathologic evaluation of the most likely route of metastasis in the thyroid. Our patient was supposed to have lymphogenous metastasis in view of unilateral lymphadenopathy and the obvious peritoneal and mediastinal lymphatic vessel infiltration.

The main method of treatment for metastatic thyroid cancers usually involves radiotherapy and surgery [8,11,17,23]. The role of radiation therapy is still controversial because thyroid metastases are mainly revealed as highly anaplastic carcinomas and are usually radiation-resistant, and are often rapidly fatal. Furthermore, until recently there has been no clear consensus on the election of surgical means for metastatic thyroid cancers [5,7,24]. Sadly, thyroidectomy had no remarkably beneficial effects on the outcome of our patient. On the whole, thyroid metastasis from esophageal cancer shows a poor prognosis, with reported 9-month survival after diagnosis [25].

## Conclusion

This case highlights the need for an awareness of the possibility of potential metastatic deposits in unexpected sites. A new thyroid mass with dysphagia appearing in a patient, however remote, should be evaluated for the possibility of metastasis. Whenever the histology is unusual for a thyroid primary, metastasis should be strongly considered. Although the prognosis of metastasis in the thyroid is commonly poor, patients with a single infiltrating thyroid mass may have improved quality of life and longer survival time after accurate diagnosis and proper treatment.

## Consent

Written informed consent was obtained from the son of the patient for publication of this Case report and any accompanying images. A copy of the written consent is available for review by the Editor-in-Chief of this journal.

## Abbreviations

ITM: intrathyroid metastases; SCC: squamous cell carcinoma.

## Competing interests

There is no financial relationship that might lead to a conflict of interest in relation to the manuscript.

## Authors’ contributions

ZXH performed the surgery and carried out the initial conception; CED and CP wrote the manuscript; YXQ, YYL and CCZ helped to revise the manuscript; JXH helped collecting references. All authors read and approved the final manuscript.

## References

[B1] Berge T Lundberg S Cancer in Malmo 1958–1969. An autopsy study Acta Pathol Microbiol Scand Suppl 1977 1 235 269649

[B2] Nakhjavani MK Gharib H Goellner JR Van Heerden JA Metastasis to the thyroid gland. A report of 43 cases Cancer 1997 79 574 578 10.1002/(SICI)1097-0142(19970201)79:3<574::AID-CNCR21>3.0.CO;2-# 9028370

[B3] Pillay SP Angorn IB Baker LW Tumour metastasis to the thyroid gland S Afr Med J 1977 51 509 512 854830

[B4] Ericsson M Biorklund A Cederquist E Ingemansson S Akerman M Surgical treatment of metastatic disease in the thyroid gland J Surg Oncol 1981 17 15 23 10.1002/jso.2930170104 7230827

[B5] Czech JM Lichtor TR Carney JA Van Heerden JA Neoplasms metastatic to the thyroid gland Surg Gynecol Obstet 1982 155 503 505 7123465

[B6] McCabe DP Farrar WB Petkov TM Finkelmeier W O'Dwyer P James A Clinical and pathologic correlations in disease metastatic to the thyroid gland Am J Surg 1985 150 519 523 10.1016/0002-9610(85)90167-9 4051119

[B7] Yoshida A Imamura A Tanaka H Hirano M Kamma H Ueno E Ushio H Aiyoshi Y Soeda S A case of metastasis from gastric cancer to the thyroid gland Jpn J Surg 1989 19 480 484 10.1007/BF02471632 2810962

[B8] Chen H Nicol TL Udelsman R Clinically significant, isolated metastatic disease to the thyroid gland World J Surg 1999 23 177 180 discussion 181 10.1007/PL00013162 9880428

[B9] Shuangshoti S Primary carcinomas of esophagus and bronchus with presentation simulating primary carcinoma of thyroid gland J Med Assoc Thai 1982 65 38 44 6153040

[B10] Yamada T Tatsuzawa Y Yagi S Fujioka S Kitagawa S Nakagawa M Minato H Kurumaya H Matsunou H Lymphoepithelioma-like esophageal carcinoma: report of a case Surg Today 1999 29 542 544 10.1007/BF02482349 10385369

[B11] Basu S Nair N Borges AM Squamous cell carcinoma of esophagus masquerading as solitary thyroid nodule Indian J Cancer 2005 42 205 207 16391440

[B12] Cumbo-Nacheli G De Sanctis JT Chung MH Proximal esophageal adenocarcinoma presenting as a thyroid mass: case report and review of the literature Thyroid 2007 17 267 269 10.1089/thy.2006.0293 17381361

[B13] Ok E Sozuer E Thyroid metastasis from gastric carcinoma: report of a case Surg Today 2000 30 1005 1007 10.1007/s005950070021 11110395

[B14] Ihn MH Kim YJ Kim JJ Cho JY Jin SY A case of thyroid metastasis originating from early gastric cancer J Korean Med Sci 2009 24 1230 1233 10.3346/jkms.2009.24.6.1230 19949691 PMC2775883

[B15] Lee HC Chen FF Lo CC Wang CJ Lo WC Luh SP Metastasis of gastric carcinoma to the thyroid and lung: a case report and review of literature J Zhejiang Univ Sci B 2010 11 542 546 10.1631/jzus.B0900378 20593521 PMC2897026

[B16] Poiana C Carsote M Ardeleanu C Terzea D Avramescu ET Neamtu MC Miulescu RD The value of the immunohistochemistry in a case of gastric neuroendocrine tumor and thyroid metastasis Rom J Morphol Embryol 2011 52 187 192 21424054

[B17] Wychulis AR Beahrs OH Woolner LB Metastasis of Carcinoma to the Thyroid Gland Ann Surg 1964 160 169 177 10.1097/00000658-196408000-00001 14209716 PMC1408803

[B18] Kihara M Yokomise H Yamauchi A Metastasis of renal cell carcinoma to the thyroid gland 19 years after nephrectomy: a case report Auris Nasus Larynx 2004 31 95 100 10.1016/j.anl.2003.09.002 15041062

[B19] Cichon S Anielski R Konturek A Barczynski M Cichon W Metastases to the thyroid gland: seventeen cases operated on in a single clinical center Langenbecks Arch Surg 2006 391 581 587 10.1007/s00423-006-0081-1 16983577

[B20] Huang TY Assor D Primary squamous cell carcinoma of the thyroid gland: a report of four cases Am J Clin Pathol 1971 55 93 98 5099785 10.1093/ajcp/55.1.93

[B21] Simpson WJ Carruthers J Squamous cell carcinoma of the thyroid gland Am J Surg 1988 156 44 46 10.1016/S0002-9610(88)80169-7 3394892

[B22] Fujita T Ogasawara Y Doihara H Shimizu N Rectal adenocarcinoma metastatic to the thyroid gland Int J Clin Oncol 2004 9 515 519 10.1007/s10147-004-0428-y 15616884

[B23] Mirallie E Rigaud J Mathonnet M Gibelin H Regenet N Hamy A Bretagnol F de Calan L Le Neel JC Kraimps JL Management and prognosis of metastases to the thyroid gland J Am Coll Surg 2005 200 203 207 10.1016/j.jamcollsurg.2004.10.009 15664095

[B24] Murakami S Yashuda S Nakamura T Mishima Y Iida H Okano H Nakano M A case of renal cell carcinoma with metastasis to the thyroid gland and concomitant early gastric cancer Surg Today 1993 23 153 158 10.1007/BF00311234 8467161

[B25] Lin JD Weng HF Ho YS Clinical and pathological characteristics of secondary thyroid cancer Thyroid 1998 8 149 153 10.1089/thy.1998.8.149 9510123

